# Screening for neuropathic pain in patients with sickle cell disease: is a single assessment scale sufficient?

**DOI:** 10.1186/s13023-019-1082-9

**Published:** 2019-05-14

**Authors:** Fabricio Dias Antunes, Cidson Leonardo Silva Junior, Karine Santos Cerqueira, Maira do Livramento Faro, Rosana Cipolotti

**Affiliations:** Federal University, Av. Beira Mar, 2016, apto 402, Aracaju-Sergipe, Brazil

**Keywords:** Sickle cell disease, Neuropathic pain, PDQ, LANSS, DN-4

## Abstract

**Background:**

The objectives of this study were to delineate the clinical-epidemiological profile of patients with neuropathic pain (NP) in the groups of SCD patients, from each of the three questionnaires used DN-4, painDETECT - PDQ, LANSS and to compare these three questionnaires in NP evaluation in SCD carriers. This cross-sectional study evaluated 83 patients with symptomatic SCD, aged 14 years or older. Clinical and laboratory data were extracted from the patients’ charts and from information obtained from the patients during the interview before the application of the questionnaire. The calculations were performed using the statistical software Epi InfoTM 7. Pearson’s correlation coefficient was used to compare the neuropathic pain evaluation scales with the software BioEstat 5.3.

**Results:**

The use of two or more questionnaires may increase the suspicion of NP in patients with SCD and, with a confirmed diagnosis, adequate treatments will benefit patients.

## Background

Sickle cell disease (SCD) is the most prevalent genetic disorder in the world, affecting about 100,000 individuals in the United States of America, or one in 500 black newborns [1–3].

SCD is characterized by recurring and severe episodes of acute pain due to occlusive vessel crises. Severe acute pain episodes are caused by tissue ischemia, resulting from the microcirculation occlusion [3]. They occur with variable intensity, with an average frequency of one to three times per year, and either disappear spontaneously or after treatment [4, 5].

Faced with the constant recurrence of acute pain episodes, there is a possibility for chronic pain (CP) to develop. Despite many studies on CP in adults with SCD in the literature, the underlying mechanisms are still not well understood [6–10]. Eventually, the pain syndrome may not be directly related to tissue injury, manifesting as a continuous and persistent sensation associated with hyperalgesia and allodynia, due to central or peripheral nervous sensitization mechanisms [11]. The CP in SCD carriers, besides raising the costs of the treatment, adds morbidity to patients already affected by frequent and serious clinical intercurrences. Neurological or psychiatric disorders, such as sleep disturbances, depression, and anxiety are quite common among patients with CP [12].

The International Association for the Study of Pain in 2011 defined neuropathic pain (NP) as “pain caused by an injury or disease of the somatosensory system” [13]. NP, a subtype of CP, is defined as a direct consequence of somatosensory system damage, having a prevalence of 7 to 8% in Europe [14, 15]. It is characterized by pain in the absence of a noxious stimulus and may be spontaneous (continuous or paroxysmal) or evoked by sensory stimuli (allodynia), a situation in which a pain response is triggered with a slight touch on the skin.

NP is associated with a variety of sensory alterations, e.g., lost or increased sensitivity, reflecting the absence of a standard clinical model in these cases [16, 17].

The neurobiological mechanisms that trigger NP, as well as the best strategies for measuring this kind of pain, are still not well understood, which represents a major problem in clinical practice [18]. The activation of sensory pathways in pain crises may serve as a triggering factor for NP and ischemia, caused by vessel-occlusion, and may lead to peripheral nervous system injury and NP [18]. Preclinical research suggests a possible mechanism related to NP, largely due to neuronal interactions with immune cells [19–21].

Studies have shown a prevalence of 20 to 25% of NP in patients with SCD, especially in women and among older patients when compared to younger patients within the samples of these studies [6, 8, 22, 23]. Patients often report their pain as burning, tingling, or pinching, which are indicative of NP [24, 25]. In general, treatment against NP shows modest effectiveness with currently available drugs [26, 27].

Diagnosing NP is not an easy task, however, the diagnosis is indispensable in order to establish the correct and effective treatment [28–32]. If NP is suspected, the diagnosis should be made based on anamnesis and physical examination data, associated with pain scales and evaluation questionnaires [8, 33, 34].

Several validated instruments are available for assessing NP. The classic DN-4 contains ten items: the first seven, called interview-DN-4, evaluate sensory characteristics, whereas the remaining three items detect signs on physical examination (hypoesthesia to the touch, hypoesthesia to needle bite and allodynia) [35]. A simple screening tool with a positive predictive value, high sensitivity and specificity is painDETECT (PDQ) which contains seven items each of which has a value ranging from 0 to 5 totaling a maximum of 35 points. Others two more points may arise if there is irradiation in that neuropathic pain mentioned and a last point depending on the characteristic of the pain reported [36]. Applying this screening tool takes about five minutes and refers to the painful experiences that the patient has had in the last four weeks [36]. Although it was developed to detect NP in patients with lower back pain, it has already been validated in patients with SCD and associated with NP [23]. LANSS, an acronym from the English version of “Leeds Assessment of Neuropathic Symptoms and Signs”, was tested and validated in several settings with sensitivity and specificity of 82–91% and 80–94%, respectively [37]. It is composed of five items about symptoms and two items about clinical examination that include an evaluation of allodynia and any alteration of the sensation threshold to needle stimulation [37]. The subjective items present yes or no answers. The negative response is zero, and the positive responses range from 1, 3, and 5 points. In both items of the physical examination when there is no change it is equal to zero and when there is change the value can be 3 or 5. The total score can reach a maximum of 24 points [37].

Our hypothesis is that associating more than one NP screening questionnaire is fundamental to detect it in SCD patients. Thus, the objectives of this study were to delineate the clinical-epidemiological profile of patients with neuropathic pain in the groups of SCD patients, from each of the three questionnaires used (DN-4, LANSS and PDQ), and to compare these three questionnaires in NP evaluation in SCD carriers.

## Method

Patients were recruited from an university outpatient clinic, which is a regional reference for SCD treatment. The patients came mostly from the state of Sergipe, but some are from municipalities in the states of Bahia and Alagoas. The three states are in the northeastern region of Brazil. Patients are treated in a standardized manner according to national and international protocols for the use of symptomatic drugs, red blood cell transfusions indication, hydroxyurea and iron chelation therapy.

This study was approved by the Human Research Ethics Committee of the Federal University of Sergipe (CAAE: 46774515.0.0000.5546). The Informed Consent Form was signed by the patient or the legal guardian in the case of a minor. All methods were performed in accordance with the relevant guidelines and regulations.

This cross-sectional study was conducted between July 2015 and December 2017 and evaluated patients with symptomatic SCD confirmed by hemoglobin electrophoresis with a minimum of 14 years-old which was the lowest age of an individual undergoing NP assessment through PDQ thus the same age was restricted for the other instruments used to avoid discrepancies [36].

The tools used in this study for NP detection were the DN-4, PDQ and LANSS scales [35–37]. The questionnaires were directly applied and the patients responded directly with a pencil or pen. Clinical and laboratory data were extracted from the patients’ charts and from information obtained from the patients during the interview before the application of the questionnaire. The data collected included name, address, age, gender, number and date of intense painful crises in the last month, hemogram results and reticulocyte counts collected during the three months prior to the evaluation in the absence of acute symptoms, medications used, previous personal history of depression, places of greater intensity of the pain. The scales used in the study were applied according to the norms presented by the authors. Each positive item in DN-4 corresponds to a point and the cut-off point for NP is 4 [35]. The PDQ questionnaire results in a total score between 0 and 38 (≥ 19 = NP, 13–18 = probable NP, ≤ 12 = absence of NP) (23; 34). In LANSS a cut-off point of 12 (out of a possible total of 24) indicates NP [37].

Chi-square or Fisher’s exact test was applied to compare the groups with and without NP as a function of the variables: adolescents (14 to 18 years) versus adults (19 to 34 years), gender and use of hydroxyurea. Average values of hemoglobin, reticulocytes, and age were compared in the groups with and without NP using ANOVA or Kruskal-Wallis tests, with values of *p* <  0.05 being considered significant. All calculations were performed using the statistical software Epi InfoTM 7. Pearson’s correlation coefficient was used to compare the neuropathic pain evaluation scales with the software BioEstat 5.3, classifying the correlation as perfect (r = 1), strong (r > 0.75), moderate (r > 0.5), weak (r <  0.5) and non-existent (r = 0). The level of significance for the test was 5%, considering values of *p* < 0.05.

## Results

The studied population corresponded to 554 individuals with symptomatic SCD. Applying the inclusion criteria (absence of a pain crisis or blood transfusion in the last month and age > 14 years), a sequential sample of 83 patients was obtained. No patient refused to participate, and all of them answered the questionnaires appropriately. The average age was 20.6 years (SD = 4.9, range 14–34) and 50.6% were male. The rest of the demographics, clinical events, and laboratory parameters are shown in Table 1.

**Table 1:** Clinical profile of patients with sickle cell anemia evaluated for investigation of NP through the DN-4, LANSS and painDETECT scales

Variables	Patients (*n* = 83)		
	DN-4	LANSS	painDETECT
**Prevalence of NP** ** (%)**	32.5	26.5	19.3
**Average age in years ± SD ** **(interval)**	20.6 ± 4.9 (14–34)		
with NP	23.2 ± 5.0 (14–34)	22.1 ± 4.3 (14–29)	24.7 ± 5.1 (15–34)
without NP	19.4 ± 4.4 (14–32)	20.1 ± 5.1 (14–34)	19.7 ± 4.4 (14–32)
**NP by age groups**	*p* < 0.01	*p* < 0.05	*p* < 0.05
Teenagers (14–18 years old)	18.5%	18.2%	12.5%
Adults (19–34 years old)	81.5%	81.8%	87.5%
**Male**		50.6%	*p* > 0.05
**Use of Hydroxyurea**		49.4%	*p* > 0.05
with NP	39%	34.1%	14.6%
without NP	61%	65.9%	85.4%
**Average of values ± SD**			
**Hemoglobin** **(g/dl)**	*p* = 0.5	*p* = 0.6	*p* = 0.02
with NP	9.5 ± 2.2	8.4 ± 0.9	7.9 ± 1.1
without NP	8.8 ± 1.4	9.0 ± 1.6	9.3 ± 1.5
**Reticulocytes** **Count** **(%)**	*p* = 0.6	*p* = 0.9	*p* = 0.01
with NP	10.8 ± 5.6	10.0 ± 6.9	13.8 ± 5.0
without NP	9.2 ± 5.1	9.5 ± 5.0	7.7 ± 4.2
**Number of self-reporting of depression**	*p* = 0.6	*p* = 0.01	*p* = 0.5
with NP	2	4	0
without NP	3	1	5
**Treatment for CP**	0 (0%)		

All patients reported mild pain in the last month, treated at home with non-opioid analgesics for oral administration according to the protocol of the service, but denied pain crises of a disabling nature or leading to the use of opioid analgesics and / or hospitalization during this period. The most frequent sites of pain in descending order were dorsal /lumbar region (65%), abdomen (10.8%), legs (7.2%), hip (6.0%), head (4.8%), feet (2,4%), joints (2.4%) and arms (1.2%).

Table 1 shows the prevalence of NP detected by questionnaire used. Age was correlated positively with NP, with higher rates among adults in relation to adolescents in all scales (Table 1). There was no significant difference between genders with regard to the presence or absence of neuropathic pain (*p* > 0.05; Chi-square).

None of the scales showed a positive association between patients using hydroxyurea and the presence of NP (Table 1). There was a significant difference between the averages of hemoglobin or reticulocytes in relation to the presence of NP in the PDQ questionnaire. An association was observed between the presence of sensory alterations identified through physical examinations of the LANSS and DN-4 scales and final score corresponding to NP (*p* < 0.01; Fisher’s exact test) (Tables 2 and 3).

**Table 2:** Comparison between the indexes of sensory changes identified by the physical mini-examination of the DN-4 scale and the final score corresponding or not to neuropathic pain

Sensory changes by physical mini-exam (DN-4)	Neuropathic pain (DN-4)		
	No	Yes	Total
No	40	4	44
%	90.9%	9.1%	100%
Yes	16	23	39
%	41%	59%	100%
Total	56	27	83
%	67.5%	32.5%	100%

**Table 3:** Comparison between the indexes of sensory changes identified by the physical mini-examination of the LANSS scale and the final score corresponding or not to neuropathic pain

Sensory changes by physical mini-exam (LANSS)	Neuropathic pain (LANSS)		
	No	Yes	Total
No	37	1	38
%	97.4%	2.6%	100%
Yes	24	21	45
%	53.3%	46.7%	100%
Total	61	22	83
%	73.5%	26.5%	100%

Although the frequency of neuropathic pain differed when the three assessment scales were compared, it was possible to detect very similar clinical results in the research with LANSS and DN-4 (Table 1). On the other hand, the PDQ showed different results compared with the two other scales (Table 1).

Regarding the comparison between the scales, Pearson’s correlation coefficient showed a statistically significant similarity between the DN-4 and LANSS questionnaires, different from the comparisons of DN-4 and PDQ or LANSS and PDQ, as shown in Table 4.

**Table 4:** Pearson correlation in the DN-4, LANSS and PDQ scales applied in patients with Sickle Cell Disease

*n* = 83	DN-4 e LANSS	DN-4 e PDQ	LANSS e PDQ
r (Pearson)	0.7143	0.2335	0.2335
(p)	< 0.0001	0.0831	0.0831

## Discussion

This study identified variable prevalence of NP in patients with SCD after evaluation with the DN-4, LANSS and PDQ scales, as shown in Table 1. There are published studies that used the same questionnaires for diagnosing chronic diseases, including SCD, and showed this frequency diversity of NP, with wide variability of 7 to 40% [8, 15, 23, 31]. The pathophysiology of NP is related to the chronic situation of origin, which may explain the great variation in the frequency of detection. In the present study, three questionnaires were used to evaluate NP in the SCD and different values ​​were observed in the frequency identified by each questionnaire, although there was a moderate correlation LANSS and DN-4 (Table 4). This statistical similarity may be related to the presence of the physical mini-exam in those two scales, whereas the PDQ doesn’t have any objective measure, being totally dependent on the examiner. However, this frequency diversity also shows the need to use more than one rating scale for NP in order to avoid patient losses during screening. The similarity between the DN-4 and LANSS scales is noticeable but they have individually shown their particularities and it is exactly in this situation that the use of only one NP screening scale may not detect a possible carrier that would most likely be detected if two or more used for this purpose. Thus, using these two instruments, these losses could be reduced and more patients could be adequately diagnosed and treated.

It was observed that the group of patients with NP had a higher average age in all three questionnaires, and that the proportion of NP was higher among adults when compared with the group of adolescents (Table 1), which was also found in previous studies [15, 23]. The recurring acute pain of patients with NP may be a triggering factor for future chronic pain as aging occurs, but there isn’t a pathophysiological explanation compatible with this hypothesis yet. However, the detection was proven in a study of abnormalities in pain perception over time [38]. It was not possible to evaluate NP in children due to a lack of validated instruments in the literature for use in individuals under the age of 14 years.

No association was found between gender and NP in this study (Table 1), which is not consistent with findings from another study that significantly related female gender with the presence of NP in the SCD [23]. However, the same group recently reported no differences between the genders in relation to the presence of NP [6]. It is important to stress that, up to now, there wasn’t any kind of pathophysiological evidence for this type of association, reinforcing that it may have been an erroneous finding.

Patients with SCD inform that is pain the most frequent uncomfortable symptom [5]. The treatment of acute pain episodes is well established, without major modifications in the years of follow-up of patients with SCD. However, the treatment of the chronic condition of NP highlighted in this study does not have unanimously efficient therapeutic options. In this study, we observe that the NP treatment has received little attention, since none of the SCD patients who were diagnosed with NP made any use of medication or other measures to address it. This situation, related to chronic diseases, is not particular to Brazil, once many other countries have given no attention to NP [10]. A correct diagnosis is essential for adequate treatment. The use of simple screening tools, such as the questionnaire used in this study, can improve these statistics and benefit DF patients [39]. In addition, scales such as DN-4, LANSS or PDQ could gauge the results of therapeutic interventions.

There was no difference between hydroxyurea use and the presence of NP (Table 1), which is not in agreement with the finding of a previous study [15]. The protocol used in Brazil for indicating hydroxyurea for patients with SCD includes frequent painful crises. Thus, this association may reflect the intensity and frequency of painful crises as a criterion for indicating hydroxyurea, and not necessarily a “cause-effect” relationship between hydroxyurea and NP [23, 31]. In addition, the present study presents cross-sectional results, a model that doesn’t contemplate identification of causal relationships.

Associated depressive symptoms were detected through a self-report by the patient and no diagnostic questionnaire was used for psychiatric disorders. Even with this limitation, the LANSS questionnaire was able to detect a positive association between depression and the presence of NP (Table 1), whereas the other questionnaires used in this study didn’t show this association. In a previous study by the same research group, in the same population but in a different sample, using a specific psychiatric tool, it was possible to detect 34.2% of patients with symptoms suggestive of depression [40]. It is important to emphasize that none of these patients used any antidepressant medication, suggesting that patients with SCD may have undiagnosed depressive disorders.

The physical mini-exam of the LANSS and DN-4 aims to complement the rest of the questionnaire to establish evidence of NP, but a complete neurological investigation with a thorough physical examination in NP is essential [36]. According to the results of LANSS and DN-4, the vast majority of the patients in this study who didn’t have alterations to the physical examination showed no evidence of NP (Tables 2 and 3), which indicates that the physical mini-examination of these questionnaires presents a good profile for the screening of NP. According to a previous study [9], sensory alterations to the physical examination reinforce the diagnosis of NP, but the absence of these findings excludes it.

A systematic review reinforced the importance of screening questionnaires for NP detection to avoid false-positive diagnoses [7]. It is known that patients with different diseases can be better evaluated through a certain scale when compared to others [36]. In this article, it was observed that the DN-4 and LANSS scales show similar results when applied to patients with SCD compared to the PDQ scale data. One justification for this finding may be the impact of the results of the physical mini-exam on the DN-4 and LANSS scores, which do not exist in the PDQ.

In addition to negatively impacting on the quality of life and psychological well-being, NP it interferes on sleep quality and activities of daily living since. Sensations as loss or reduction of sensibility, shock-type pain, hyperalgesia or allodynia will accompany patients throughout life and may be worse if the trigger of the nerve damage that caused this chronic pain has not been controlled independent of the underlying disease. In the cases of SCD these patients still coexist with another pain type of acute and recurrent character that also interferes enough in the quality of life of these individuals, limiting them to each day [9, 41, 42]. The earlier diagnosis of NP, the earlier therapy will be initiated and consequently there will be an improvement in the quality of life of patients with SCD.

## Limitations

The limitation of this study is the cross-sectional design. So prospective studies will be required to verify if there is a cause-effect relationship between SCD and NP.

## Conclusion

Therefore, it is concluded that it was possible to identify frequencies of 19.3, 26.5 and 32.5% of patients with NP through the PDQ, LANSS and DN-4 scales, respectively, affecting adults more frequently. Using more than one NP evaluation scale amplifies the detection rate. Sensory investigation through directed physical examination increases the identification capacity of NP patients even in the absence of suggestive anamnesis. None of the patients identified had any type of NP treatment. Thus, the use of questionnaires may increase the suspicion of NP in patients with SCD and, with a confirmed diagnosis, adequate treatments will benefit the patients.
